# Identification of pyroptosis-related long non-coding RNAs with prognosis and therapy in lung squamous cell carcinoma

**DOI:** 10.1038/s41598-022-15373-6

**Published:** 2022-07-01

**Authors:** Yi Zhang, Yuzhi Wang, Xiaoqing Yin, Yi Huang

**Affiliations:** 1 Shengli Clinical Medical College of Fujian Medical University, Fuzhou, 350001 Fujian People’s Republic of China; 2 Department of Laboratory Medicine, People’s Hospital of Deyang City, Deyang, 618000 Sichuan People’s Republic of China; 3 Fujian University of Traditional Chinese Medicine, Fuzhou, 350001 Fujian People’s Republic of China; 4 Department of Clinical Laboratory, Fujian Provincial Hospital, Fuzhou, 350001 Fujian People’s Republic of China; 5 Center for Experimental Research in Clinical Medicine, Fujian Provincial Hospital, Fuzhou, 350001 Fujian People’s Republic of China; 6 Central Laboratory, Fujian Provincial Hospital, Fuzhou, 350001 Fujian People’s Republic of China

**Keywords:** Lung cancer, Prognostic markers, Genetic markers, Non-coding RNAs, Risk factors, Data mining

## Abstract

Pyroptosis is a type of programmed cell death with an intense inflammatory response. Previous studies have shown that pyroptosis plays an important role in the pathogenesis and progression of lung cancer. However, the prognostic value and drug targets of pyroptosis-related lncRNAs in lung squamous cell carcinoma (LSCC) have never been studied. In the present study, we identified 1468 pyroptosis-related lncRNAs in LSCC by performing Pearson correlation analysis between the pyroptosis-related genes and the lncRNAs from The Cancer Genome Atlas (TCGA) and Gene Expression Omnibus (GEO) database. The whole set was divided into a training and a test set with a 1:1 ratio. Univariate Cox regression and least absolute shrinkage and selection operator (LASSO) analyses were conducted to establish an 11 multilncRNA signature in the three sets. The signature divided LSCC patients into the low-risk and the high-risk groups. Kaplan–Meier analysis and receiver operating characteristic (ROC) indicated that the prognostic signature had a promising predictive capability for LSCC patients. Besides, the association of microenvironment and immunotherapy response with signature was also analyzed. Moreover, 28 potential compounds targeting signature were screened as possible drugs to treat LSCC. Finally, a nomogram model was constructed to offer the quantitative prediction and net benefit for the prognosis of LSCC patients. In conclusion, the 11 pyroptosis-related lncRNAs and their signature may be promising prognostic factors and therapeutic targets for patients with LSCC.

## Introduction

Lung cancer is the foremost cause of cancer-associated deaths worldwide and ranks first in terms of morbidity and mortality of all cancer types^1^. It can be classified into small cell lung cancer (SCLC) and non-small cell lung cancer (NSCLC) according to the histopathological presentation of tissues. NSCLC is further divided into lung adenocarcinoma (LUAD), lung squamous cell carcinoma (LSCC), and large-cell lung cancer (LCLC)^2^. LSCC constitutes 25–30% of all cases of lung cancer, which is primarily caused by tobacco smoking^3^. Compared with LUAD, LSCC has a poor clinical prognosis, and its 5-year survival rate is less than 15%^4^. The main methods of treating lung cancer include traditional chemotherapy, radiation therapy, targeted therapy and surgery. However, surgical resection is only feasible for treating early-stage lung cancer in most cases, and some patients do not achieve a curative response to chemotherapy and radiotherapy owing to drug resistance and severe side effects. Therefore, comprehending the molecular mechanisms underlying tumorigenesis and tumour progression to identify novel biomarkers and therapeutic targets is critical for improving early diagnosis, therapeutic effects and prognosis in patients with LSCC.

Pyroptosis, inflammation-induced programmed cell death with high specificity, is a new type of programmed cell death^5^. It involves pore formation in the plasma membrane, swelling of the cell and rupture of the cell membrane, followed by massive leakage of cytosol. Pore formation is associated with cellular death, swelling and rupture of the plasma membrane and infiltration of cytosolic contents^6,7^. Recent studies have suggested that pyroptosis plays a role in the genesis and progression of various cancers, and activating pyroptosis to treat tumours has significant therapeutic potential^8^. Several relevant studies have shown that prodrugs, such as berberine, euxanthone and alpinumisoflavone, activate pyroptosis and kill hepatic cell carcinoma cells through caspase-1-dependent pyroptosis^9–11^. DPP8 inhibitors promote pyroptotic cell death in human acute myeloid leukaemia cells and suppress tumour progression in mouse models, suggesting its potential utility as a therapeutic agent^12^. In addition, pyroptosis has recently been recognised to function as an important factor in lung cancer. In a study, the mRNA and protein levels of P53 were positively correlated with pyroptosis in patients with NSCLC, and P53 may promote pyroptosis through its direct binding and activation of NLRP3^13^. Moreover, Teng et al. reported that polyphyllin VI isolated from *Trillium tschonoskii* maxim triggers caspase-1-mediated pyroptosis in A549 and H1299 cells by activating the ROS/NF-κB/NLRP3/GSDMD signalling axis^14^. These studies indicate that pyroptosis may effectively treat lung cancer and improve patient outcomes.

Long non-coding RNAs (lncRNAs) are a class of non-coding RNA molecules that contain more than 200 nucleotides without protein-coding function^15^. They are involved in various biological behaviour processes in eukaryotes, and their abnormal expression is associated with the pathophysiological process of various tumours, including cell growth, differentiation and invasion and drug resistance^16,17^. However, the role of lncRNAs in regulating pyroptosis in lung cancer development remains to be elucidated. Therefore, pyroptosis-related biomarkers should be identified through high-throughput sequencing to guide the diagnosis, prognosis and treatment of patients with LSCC.

In this study, we identified pyroptosis-related lncRNAs using the The Cancer Genome Atlas (TCGA) database and constructed a multi-lncRNA signature via bioinformatic and statistical analyses for the prediction of survival in patients with LSCC. In addition, we investigated the relationship between the pyroptosis-related lncRNA signature and microenvironment to enhance the understanding of the occurrence and development of LSCC. In addition, we discovered candidate drugs targeting the signature to predict immunotherapy responses. Eventually, we established a nomogram for the quantitative prediction of overall survival (OS) for patients with LSCC.

## Materials and methods

### Data collection and processing

RNA-sequencing data, somatic mutation data and relevant clinical information of patients with LSCC were downloaded from TCGA Data Portal (https://portal.gdc.cancer.gov/). Patients (n = 475) were included for subsequent analysis if their follow-up time was more than 28 days and were randomly divided into the training (n = 239) and test sets (n = 236) in a ratio of 1:1. lncRNA and mRNA expression data were categorised depending on the annotations provided by the GENCODE project^18^. In addition, the independent dataset GSE81089, transcriptome profiles and clinical information of 195 tumour samples based on the platform GPL16791 were acquired from the Gene Expression Omnibus (GEO) dataset (http://www.ncbi.nlm.nih.gov/geo/). The baseline clinical characteristics of patients were collected in the Table 1. All methods were carried out in accordance with relevant guidelines and regulations.

**Table 1:** Clinical characteristics of the TCGA cohort.

Characteristics	Groups	Number (percentage)
Age	< 60	90 (19%)
	> 60	380 (81%)
Gender	Male	353 (74%)
	Female	122 (26%)
T	T1–T2	386 (81%)
	T3–T4	89 (19%)
N	N0	301 (64%)
	N1–N3	169 (36%)
M	M0	395 (96%)
	M1	16 (4%)
Stage	Stage I–II	384 (81%)
	Stage III–IV	87 (19%)
Cancer status	Tumor free	318 (71%)
	With tumor	129 (29%)

### Exploration of pyroptosis-related lncRNAs

The 33 pyroptosis-related genes were acquired from several previous studies^19–22^, which are presented in Table S1. To identify the potential lncRNAs associated with pyroptosis, we conducted Pearson’s correlation analysis on pyroptosis-related genes and lncRNAs. The thresholds were set as follows: correlation coefficient (|R|) > 0.4 and *P*-value < 0.001.

### Identification and validation of a pyroptosis-related lncRNA prognostic signature for LSCC

To screen pyroptosis-related lncRNAs associated with survival, univariate Cox regression was performed with *P* < 0.05 as the criteria. The least absolute shrinkage and selection operator (LASSO) regression with fivefold cross-validation analysis was employed to further filter the variables from the results of the univariate Cox analysis. Subsequently, a pyroptosis-related lncRNA signature was established using multivariate Cox proportional hazards analysis. The risk score was calculated for each patient as follows: risk score = β1 (lncRNA1) × expr (lncRNA1) + β2 (lncRNA2) × expr (lncRNA2) + ⋯ + βn (lncRNAn) × expr (lncRNAn), where βn is the coefficient of lncRNAs, and expr is the expression of lncRNAs. Based on the median risk score in the training set, patients in the three sets were divided into the low- and high-risk groups. Another independent GEO cohort with 195 patients was used for validating the signature. The Kaplan–Meier log-rank test was performed to estimate the survival difference between the high- and low-risk groups. Eventually, time-dependent receiver operating characteristic (ROC) curves were used to evaluate the prognostic value of the signature. We obtained the expression of lncRNAs in the signature from GSE81089 and calculated the risk score of each patient using the same method in the TCGA cohort. Moreover, we screened for some lncRNA signatures from previous studies and compared their performance with that of the signature established in this study by estimating the AUC values in ROC curves^23,24^.

### Functional enrichment analysis

Differentially expressed genes between the high- and low-risk groups were identified using the ‘limma’ package in R. The cut-off was set at false discovery rate (FDR) < 0.05 and |log2foldchange (FC)|≥ 1. Gene Ontology (GO)^25^ and Kyoto Encyclopedia of Genes and Genomes (KEGG)^26^ annotations were examined using the gene set enrichment analysis (GSEA) software (version 4.1). Gene sets with more than 20 genes and less than 500 genes were included for GSEA analysis.

### Tumour microenvironment and immune response analysis

The mutation data were processed and viewed using the ‘maftools’ package in R. The tumor mutation burden (TMB) of patients was calculated from tumor-specific mutation genes^27^. The estimation of stromal and immune cells in malignant tumor tissues using expression data (ESTIMATE) algorithm was used to estimate immune scores (immune, stromal and ESTIMATE scores), which reflected the ratio of stromal and immune cells in each sample. In addition, tumour purity was estimated. The Single-sample gene set enrichment analysis (ssGSEA) algorithm with the ‘SVA’ package in R was used to assess the relative abundance of 16 tumour-infiltrating immune cells and 13 immune cell functions. Potential 27 immune checkpoint genes were also obtained from previous studies. The association between tumour stemness and the signature was assessed using Spearman correlation analysis. The tumor immune dysfunction and exclusion (TIDE) algorithm was employed to predict the individual likelihood of immunotherapy response^28^.

### Screening of potential compounds targeting the signature

Genomics of Drugs Sensitivity in Cancer (GDSC) is an online database that contains data on various anti-cancer drugs based on multiple cell lines^29^. To screen for potential drugs for LSCC, we estimated half-maximal inhibitory concentration (IC50) of compounds from the GDSC database (https://www.cancerrxgene.org/) using the pRRopheticPredict function with ridge regression.

### Construction of a nomogram

To screen for independent predictors of outcomes based on the clinicopathological characteristics, we implemented univariate and multivariate Cox regression analyses parameters including age, sex, tumour location, Tumor–Node–Metastasis (TNM) stage and cancer status. *P* < 0.05 was considered statistically significant. Based on independent predictors, a nomogram was constructed to quantitatively predict the survival probability of patients with LSCC. The concordance index (Cindex) plot, calibration cures and decision curve analysis (DCA) were used to assess the predictive performance and benefit of the nomogram.

### Cell culture

A normal lung epithelial cell line (BEAS-2B) and LSCC cell lines (H226, SK-MES-1) and were purchased from the American Type Culture Collection (Manassas, Virginia, USA). SK-MES-1 cell line was cultured in a Dulbecco’s modified Eagle’s (DMEM) medium (Hyclone, Thermo Fisher Scientific, Waltham, USA) with 10% Fetal bovine serum (FBS, MilliporeSigma, Burlington, MA, USA), and BEAS-2B and H226 cell lines were cultured in Roswell Park Memorial Institute (RPMI) 1640 medium (Hyclone, Thermo Fisher Scientific, Waltham, USA) with 10% FBS. All cell lines were incubated at 37 °C, 5% CO2.

### Tissue samples

We collected 20 pairs of LSCC cancerous tissue and matched para-cancerous samples from surgical patients in the Fujian Provincial Hospital. Samples were handled by RNA preservation solution and stored at − 80 °C until RNA extraction. The ethics committee of Fujian Provincial Hospital approved this study (Ethics Approval Number K2021-11-012).

### Reverse transcription and quantitative real-time polymerase chain reaction (qRT-PCR)

Total RNA for cell lines and tissue samples was isolated using Trizol reagent (Invitrogen; Thermo Fisher Scientific, USA). Then cDNA was synthesized using Prime-Script RT Kit (Promega Corporation, Madison, USA) for reverse transcription and Promega SYBR-Green PCR Master Mix (Promega Corporation, Madison, USA) for qRT-PCR on Roche LightCycler480 II real-time PCR system (Roche, Germany). The values of Ct were calculated with the 2−ΔΔCt method and normalized to the expression levels of β-actin. All PCR tests were conducted at least three times. The primer sequences used in this study are shown in Table S2.

### Statistical analyses

All calculations and visualizations were performed using Perl and R statistical software (version 4.0.5). Differences between the groups were analysed using the Chi-square or Fisher test for categorical variables and Wilcoxon test for continuous variables. Kaplan–Meier survival curves in different groups were compared using the log-rank test. Differences were considered statistically significant at *P* < 0.05 unless specified otherwise.

### Ethics approval and consent to participate

Ethics approval was sought and approved from the Ethics Committee of Fujian Provincial Hospital (Ethics Approval Number K2021-11-012). The patients/participants provided their written informed consent to participate in this study.

## Results

### Identification of pyroptosis-related lncRNAs with significant prognostic value in LSCC

**Figure 1:**
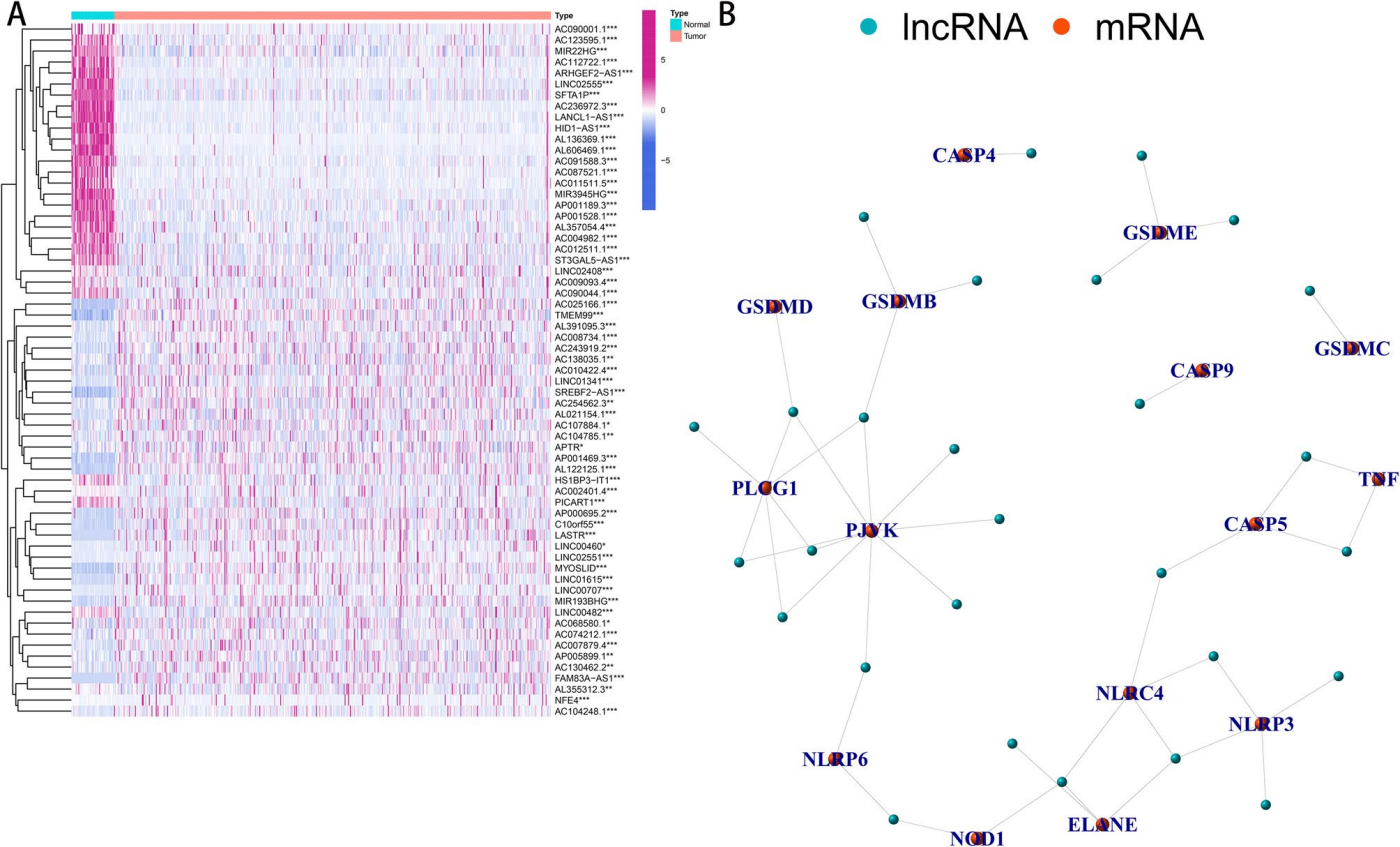
Identification of pyroptosis-related lncRNAs in LSCC patients. (**A**) Heatmap for expression of pyroptosis-related lncRNAs in normal and tumor tissue. (**B**) Gene co-expression network of candidate lncRNAs and pyroptosis-related genes. *lncRNA* long non-coding RNAs, *LSCC* lung squamous cell carcinoma.

In the expression matrix, 19,658 probes and 14,049 probes were annotated to mRNAs and lncRNAs, respectively, according to the Ensemble database (https://www.ensembl.org/). A total of 1468 pyroptosis-related lncRNAs were identified via Pearson correlation analysis of pyroptosis-related genes and lncRNAs with the standard threshold of |R|> 0.4 and *P* < 0.001. Of these pyroptosis-related lncRNAs, 63 were significantly correlated with the OS of patients with LSCC via univariate Cox regression and Kaplan–Meier analyses. A total of 63 lncRNAs were significantly differentially expressed between normal and adjacent tissues (Fig. 1A). The co-expression relationship between prognostic pyroptosis-related lncRNAs and genes is illustrated in Fig. 1B.

### Construction of the pyroptosis-related lncRNA prognostic signature

**Figure 2:**
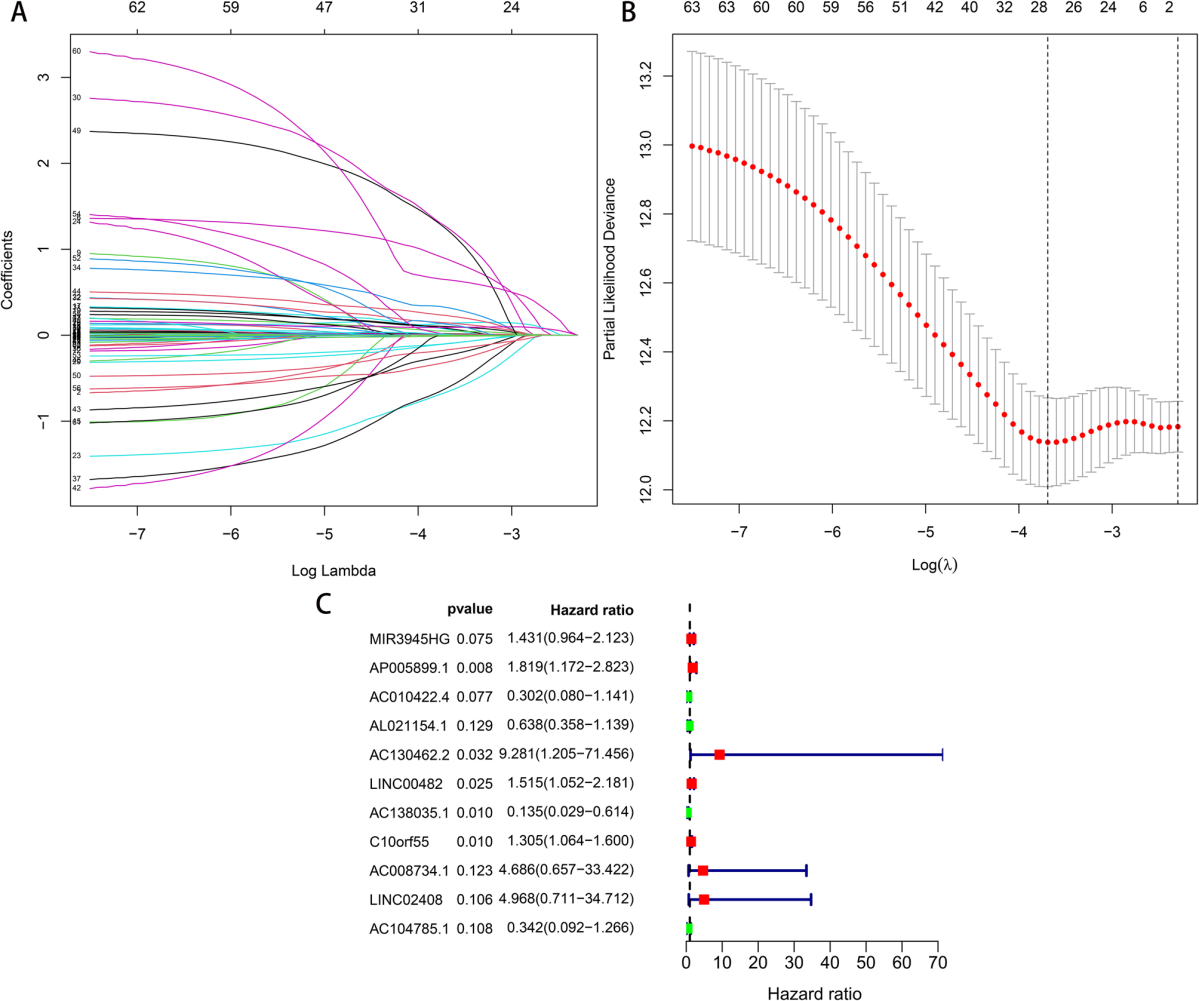
Construction of the pyroptosis-related lncRNA prognostic signature in LSCC patients. (**A**) Selection of the optimal parameter (lambda) via 5 times cross-validation. (**B**) LASSO coefficient profiles of 28 pyroptosis-related lncRNAs. (**C**) 11 lncRNAs were identified for constructing A signature by multivariate Cox ratio hazard regression analysis. *lncRNA* long non-coding RNAs, *LSCC* lung squamous cell carcinoma, *LASSO* least absolute shrinkage and selection operator.

**Figure 3:**
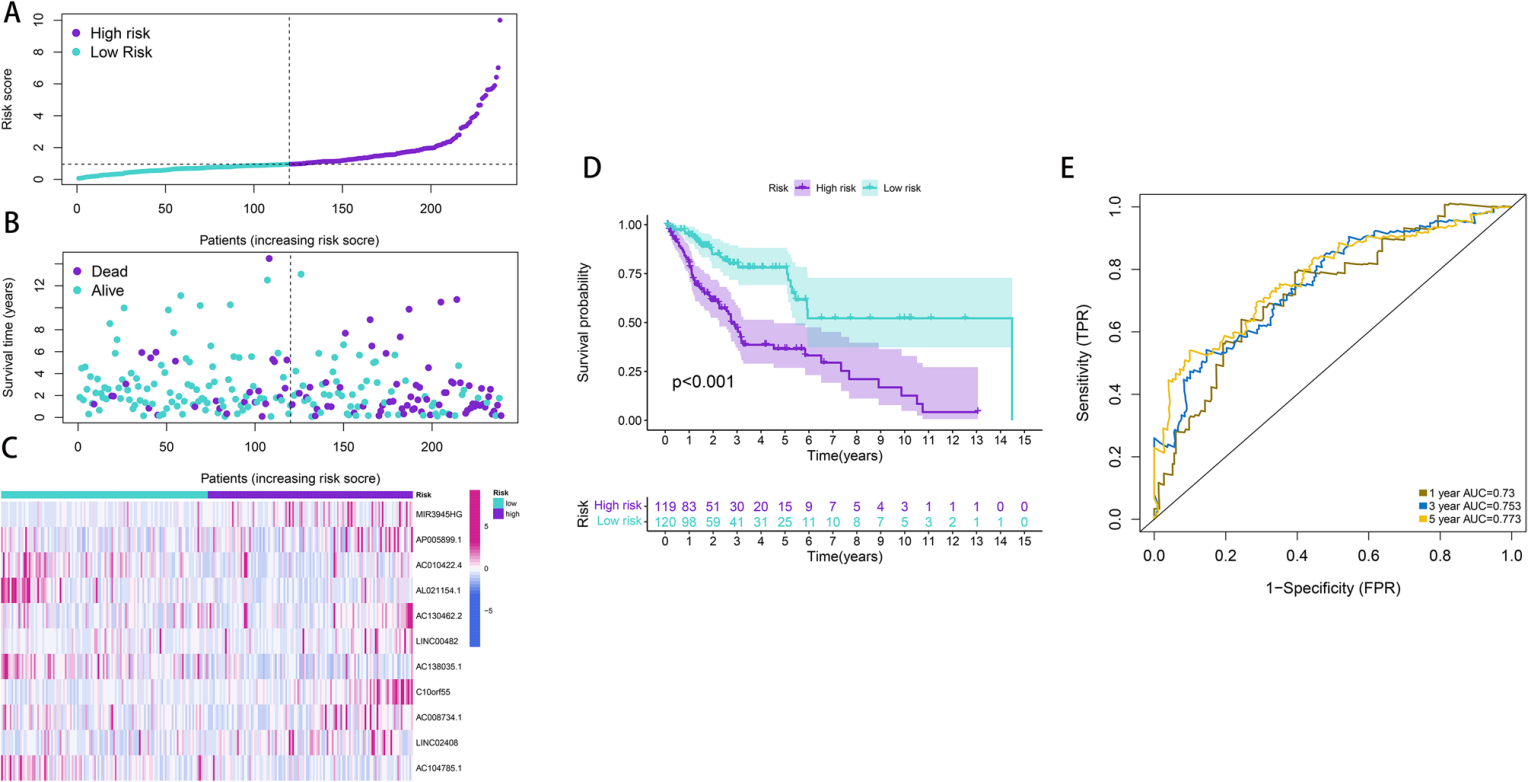
Prognostic analysis of signature in the TCGA training set. (**A**) The distribution of risk scores. (**B**) Patient distribution of survival status in the high-risk and low-risk groups. (**C**) The heatmap showing expression profiles of the 11 pyroptosis-related lncRNAs. (**D**) Survival analysis in the high-risk and low-risk groups. (**E**) ROC curve for 1, 3, and 5 year OS predictions based on the signature. *TCGA* The Cancer Genome Atlas, *lncRNA* long non-coding RNAs, *ROC* receiver operating characteristic, *OS* overall survival.

**Figure 4:**
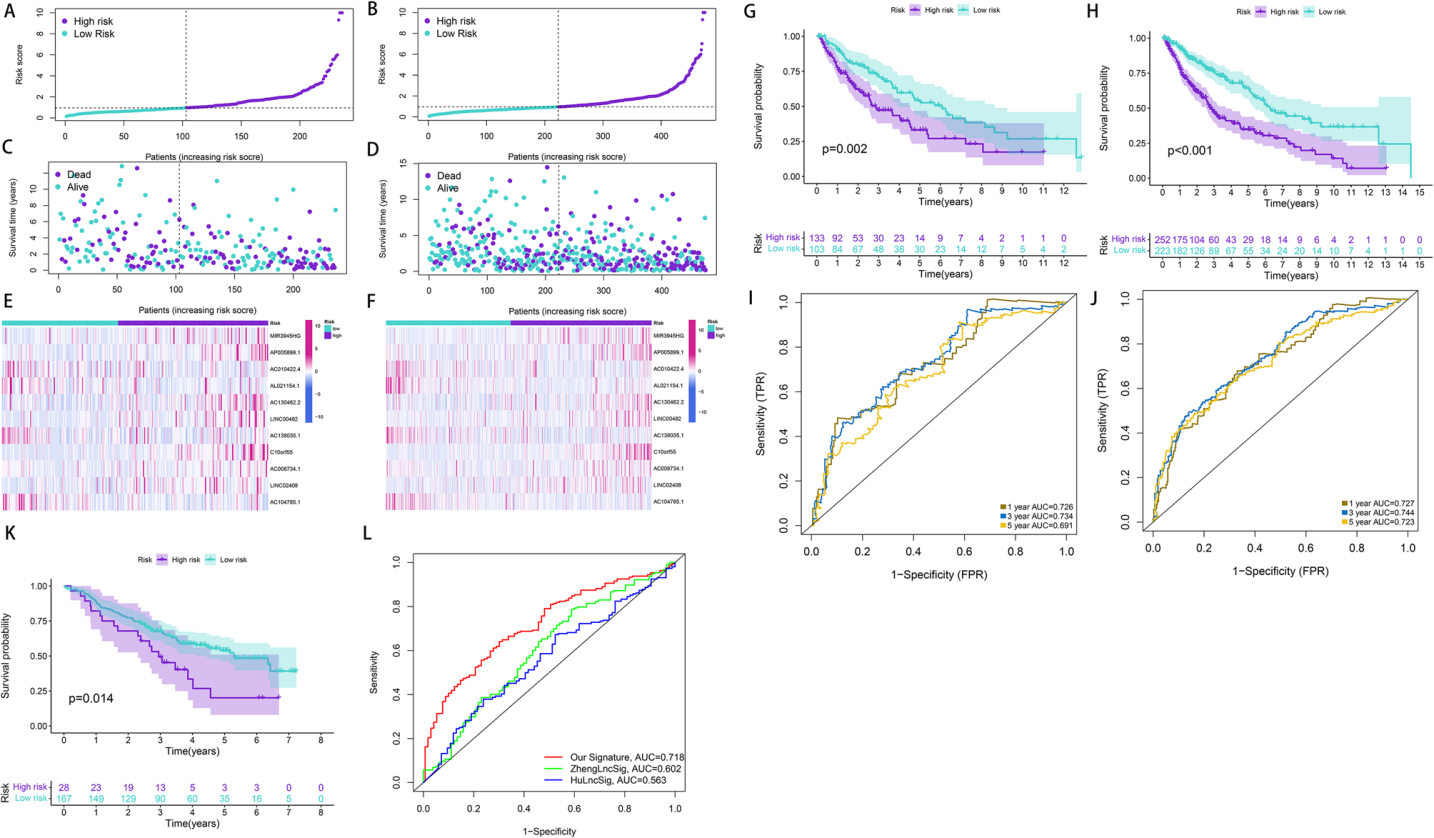
Prognostic analysis of signature in the validation cohorts. (**A,B**) The distribution of risk scores in the TCGA validation set and entire set. (**C,D**) Patient distribution of survival status in the high-risk and low-risk groups in the TCGA validation set and entire set. (**E,F**) The heatmap showing expression profiles of the 11 pyroptosis-related lncRNAs in the TCGA validation set and entire TCGA set. (**G,H**) Survival analysis in the high-risk and low-risk groups for the TCGA validation set and entire set. (**I,J**) ROC curve for 1, 3, and 5 year OS predictions based on the signature the TCGA validation set and entire set. (**K**) Kaplan–Meier analysis of high-risk and low-risk patients for GSE81089 set. (**L**) ROC curves for 5 year OS predictions by our signature and previously reported signatures. *TCGA* The Cancer Genome Atlas, *lncRNA* long non-coding RNAs, *ROC* receiver operating characteristic, *OS* overall survival.

**Figure 5:**
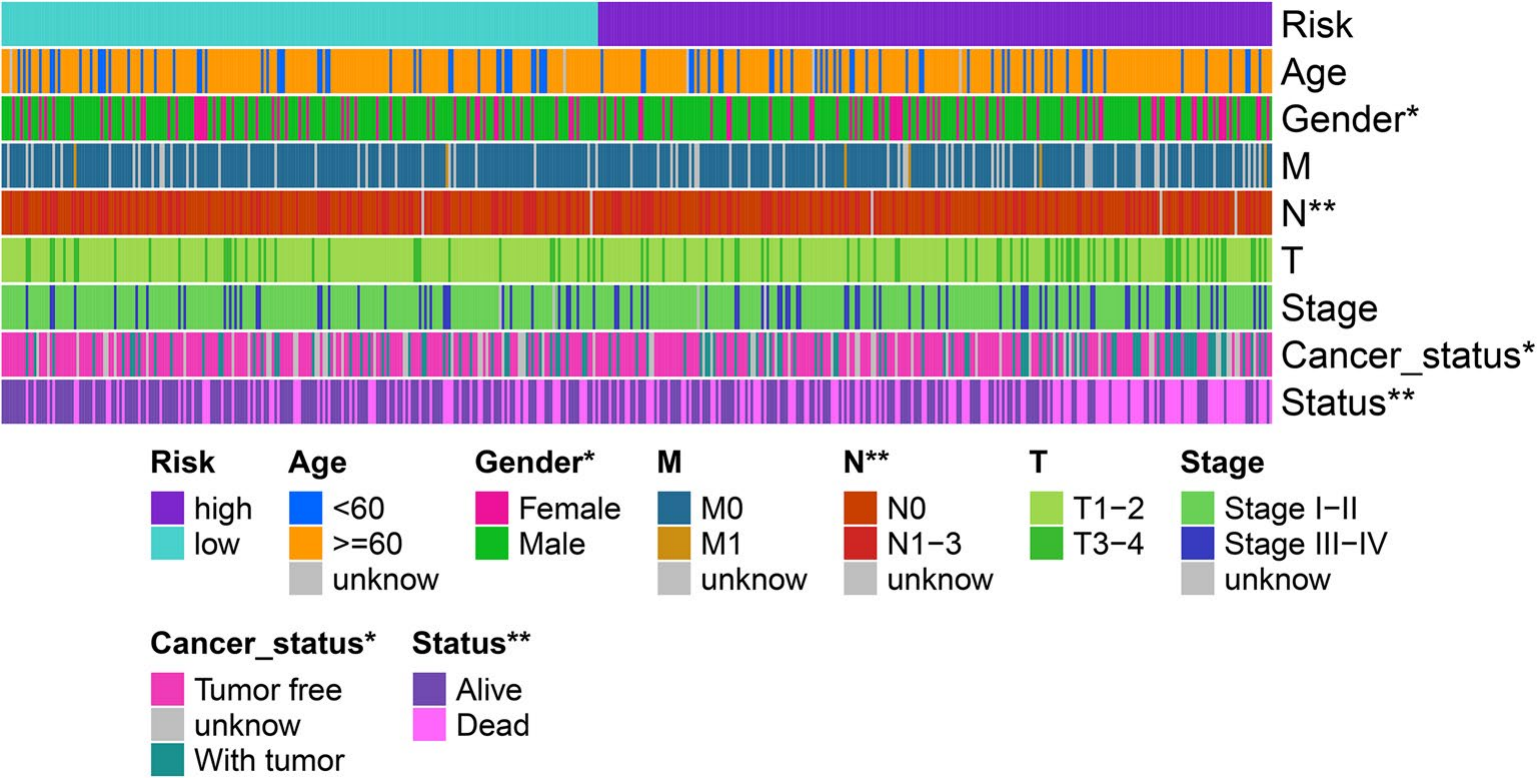
Relationship between the signature and clinicopathological features. *P < 0.05, **P < 0.01, ***P < 0.001.

**Figure 6:**
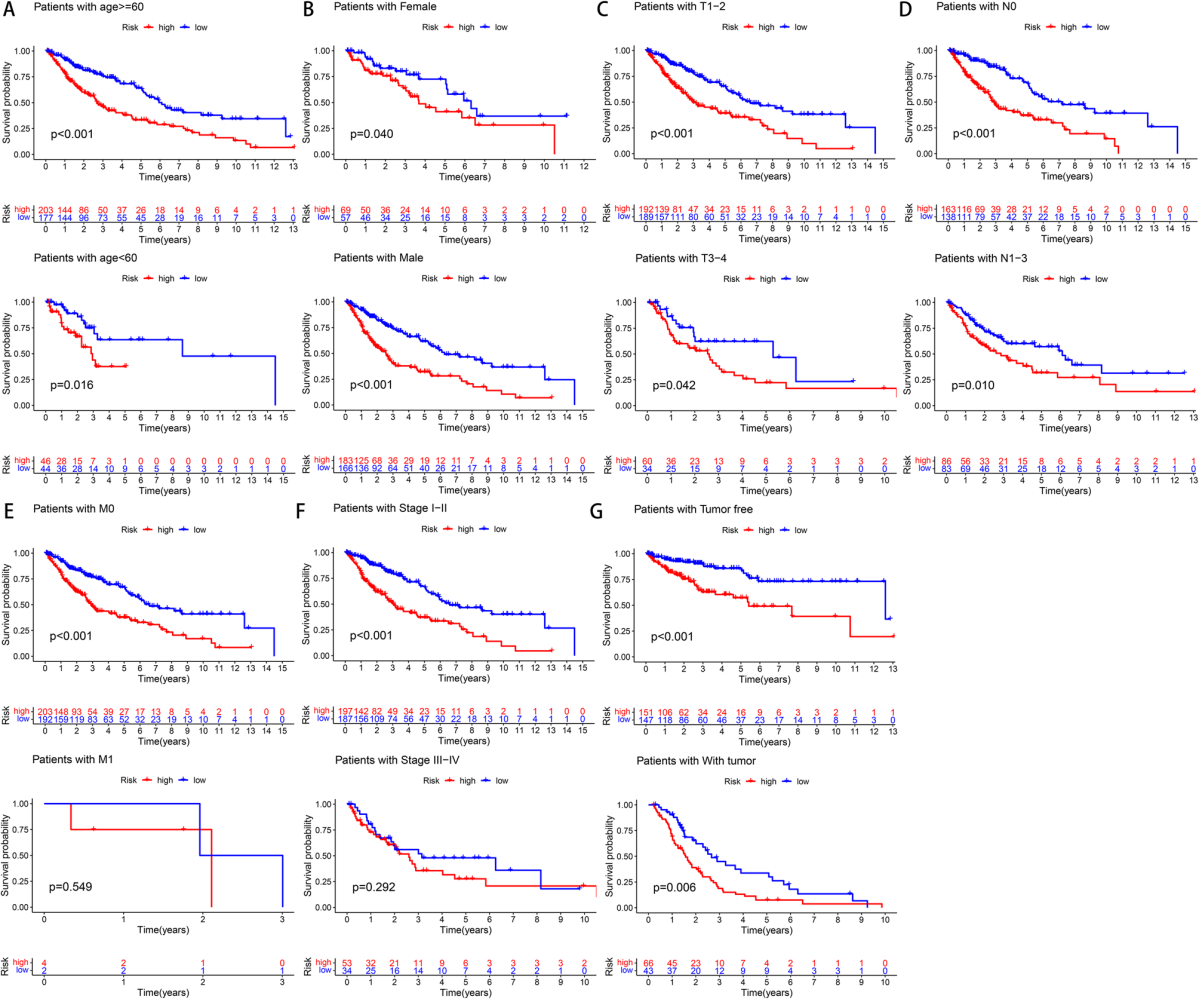
Kaplan–Meier survival curves of LSCC patients in different clinical subgroups. (**A**) Age (≤ 60 or > 60 years old). (**B**) Sex (female or male). (**C**) T (T1–2 or T3–4). (**D**) N (N0 or N1–3). (**E**) M (M0 or M1). (**F**) Stage (I–II or III–IV). (**G**) Cancer status (tumor free or with tumor). *TCGA* The Cancer Genome Atlas, *LSCC* lung squamous cell carcinoma, *TNM* tumor-node-metastasis.

First, patients were randomly divided into the training (n = 239) and test (n = 236) sets. To further enhance the reliability and utility of the signature, LASSO–Cox regression with a fivefold cross-validation analysis was used to select optimal features (Fig. 2A,B). Subsequently, 28 pyroptosis-related lncRNAs were selected for multivariate Cox proportional hazard regression analysis. Finally, we constructed a signature based on 11 pyroptosis-related lncRNAs to predict the OS of patients with LSCC in the training set (Fig. 2C). The patients were categorised into the high- and low-risk groups based on the intermediate-risk scores (Fig. 3A–C). In addition, Kaplan–Meier survival curves showed that patients in the low-risk group had better clinical outcomes than those of patients in the high-risk group (Fig. 3D). In addition, the AUC values of ROC for the signature at 1-, 3- and 5-year were 0.73, 0.753 and 0.773, respectively (Fig. 3E). To further assess the robustness and accuracy of the signature, we built the same signatures in the test and whole sets using the same algorithm and divided patients into the high- and low-risk groups based on the same cut-off values (Fig. 4A–F). Similarly, the mortality rate in the high-risk groups was higher than that in the low-risk groups in the test and whole sets (Fig. 4G,H). The AUC values for 1-, 3- and 5-year were 0.726, 0.734, and 0.691, respectively, in the test set and 0.727, 0.744 and 0.723, respectively, in the whole set (Fig. 4I,J). To verify the reliability of the signature, we established the same signature using the GSE81089 dataset according to the aforementioned formula. The survival curve revealed that the signature exhibited good performance in predicting prognosis (Fig. 4K). Moreover, we compared the survival prediction ability of the signature with two recently reported lncRNA-based signatures for LSCC using the same TCGA cohort. As depicted in Fig. 4L, the established signature with the biggest AUC value of ROC at 5-year OS had better prediction efficacy than that of ZhenglncSig and HulncSig. Furthermore, the association between the signature and clinicopathological features was analysed. The risk scores were found to be significantly correlated with sex, N stage, cancer status and survival status (Fig. 5). To demonstrate the applicability of the signature, we further performed subgroup survival analysis for the whole set using the following clinicopathological features: age, sex, TNM stage, stage and cancer status. As a result, except for M0 and stage III–IV groups, patients in the high-risk group had significantly worse outcomes than those of patients in the low-risk groups for each subgroup (Fig. 6). Collectively, these results indicate the effectiveness and robustness of the established signature in predicting the survival of patients with LSCC.

### Biological function and pathway analyses

**Figure 7:**
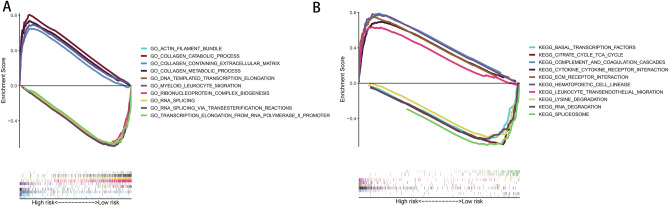
GSEA analysis between the high-risk and low-risk group in the TCGA whole set. (**A**) Signature was associated with gender, N, cancer status and status. (**A**) GO term enrichment analysis. (**C**) KEGG pathways analysis. *TCGA* The Cancer Genome Atlas, *GSEA* gene set enrichment analysis, *GO* Gene Ontology, *KEGG* Kyoto Encyclopedia of Genes and Genomes.

We performed GO and KEGG pathway analyses between the high- and low-risk groups using the GSEA algorithm. GO term enrichment analysis indicated that the high-risk group was mainly enriched in actin filament bundle, collagen catabolism pathways, collagen-containing extracellular matrix, collagen metabolism pathways and myeloid leukocyte migration, whereas the low-risk group was mainly enriched in DNA-templated transcription elongation, ribonucleoprotein complex biogenesis, RNA splicing, transcription elongation from RNA polymerase and splicing via transesterification (Fig. 7A). The results of KEGG analysis revealed that the high-risk group was involved in complement and coagulation cascades, cytokine receptor interaction, ECM receptor interaction, haematopoietic cell lineage and leukocyte transendothelial migration, whereas the low-risk group was involved in basal transcription factors, citrate cycle (TCA cycle), lysine degradation, RNA degradation and spliceosomes (Fig. 7B).

### Estimation of tumour microenvironment and immunotherapy response using the signature

**Figure 8:**
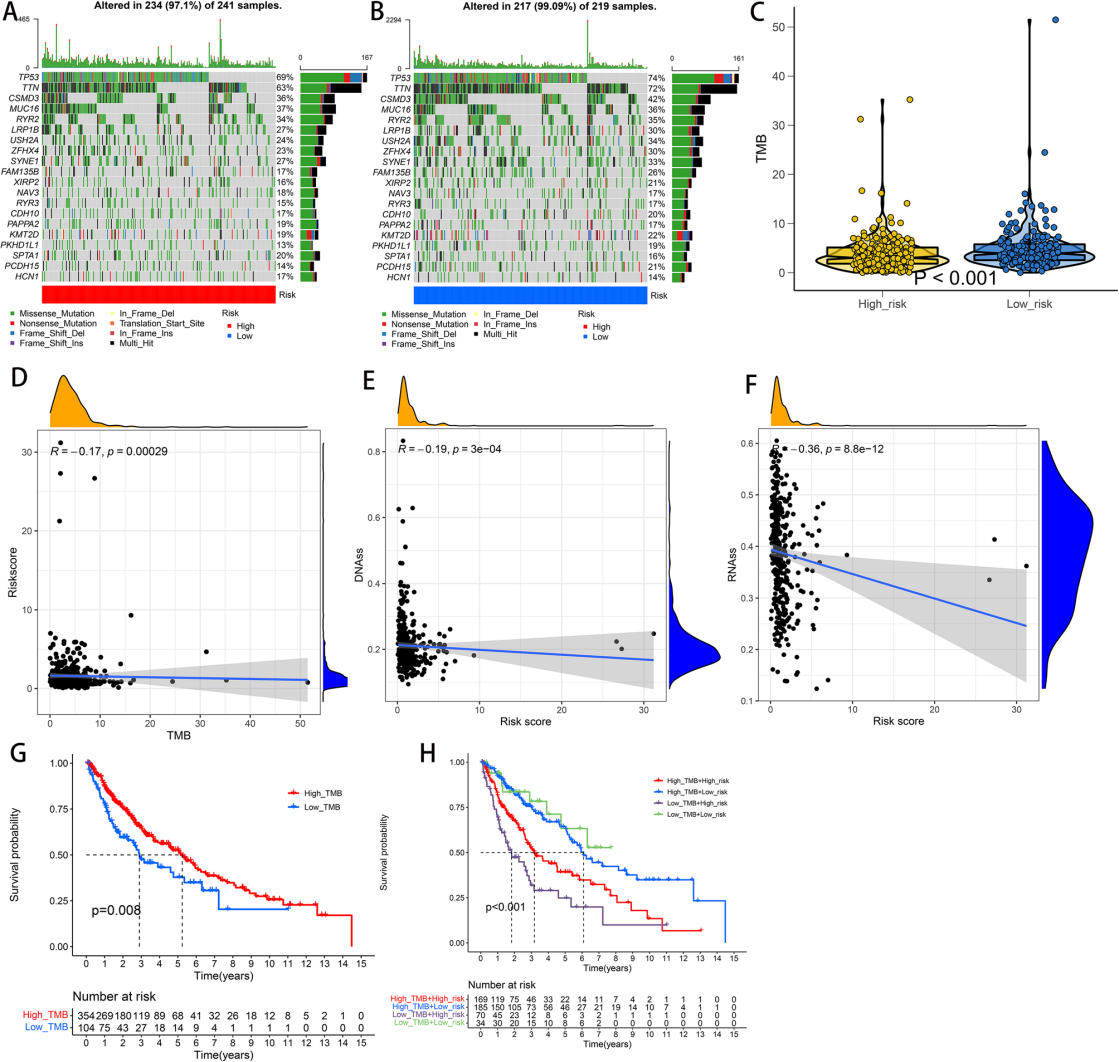
Somatic mutations analysis and the association between risk score and the tumor microenvironment in the TCGA whole set. (**A,B**) Waterfall plots showing the mutation information of the top 20 genes with the highest mutation frequency in low-risk and high-risk groups. (**C**) Distribution of TMB in two groups. (**D–F**) Relationship between risk score and TMB, DNAss and RNAss. (**G,H**) Kaplan–Meier survival curves revealed the prognostic value of TMB with or without combination with the risk score. *TCGA* The Cancer Genome Atlas, *TMB* tumor mutation burden, *DNAss* DNA stemness scores, *RNAss* RNA stemness scores.

**Figure 9:**
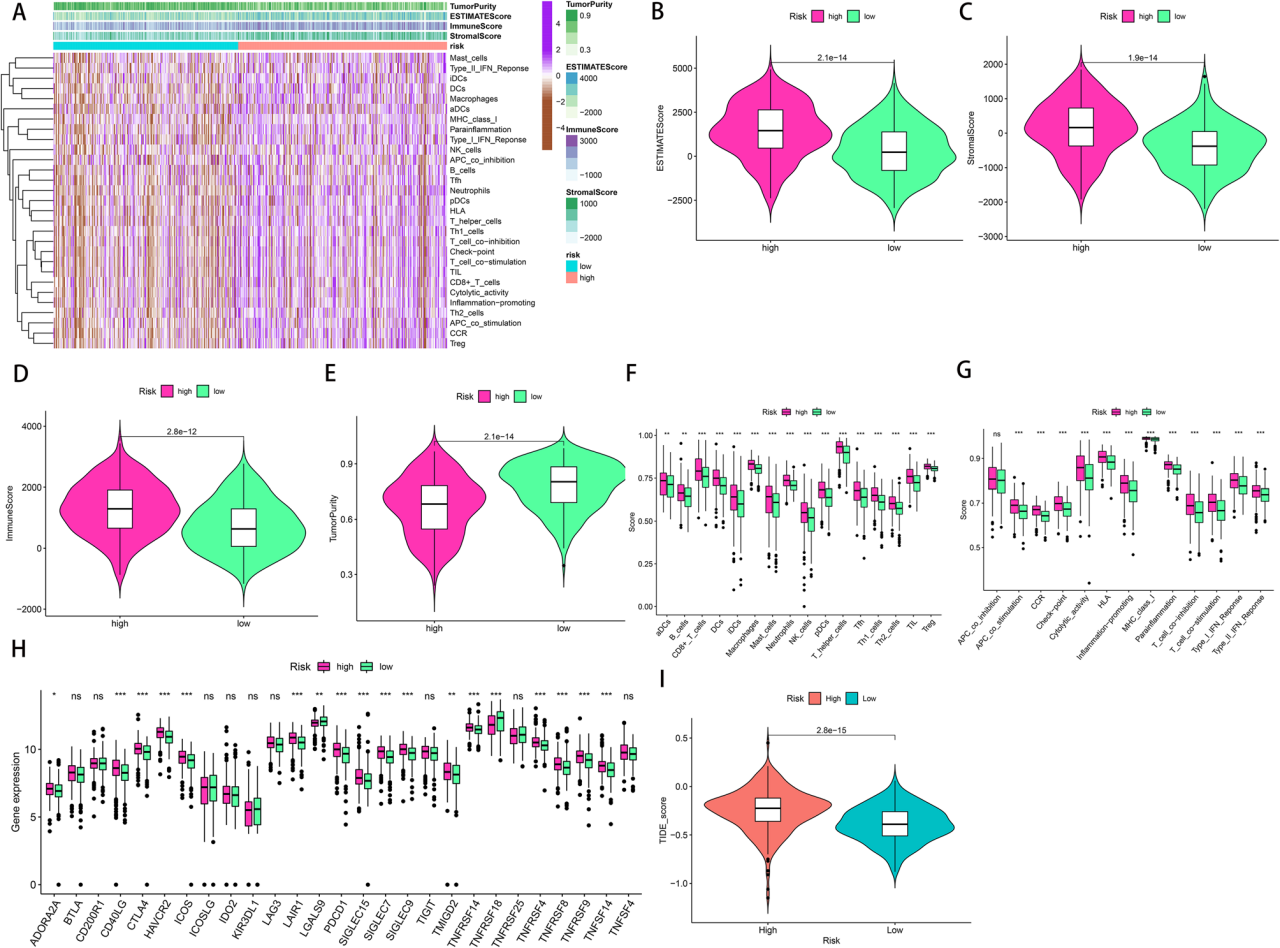
Estimation of the immune status and response to immunotherapy based on the signature in the high-risk and low-risk groups. (**A**) Heatmap of immune cell infiltration. (**B–E**) Violin plots for the immune score, stromal score, ESTIMATE score, and tumor purity. (**F–H**) Boxplots of immune cells score, immune-related functions score and immune checkpoints expression. (**I**) Prediction of immunotherapy response according to TIDE score. *ESTIMATE* estimation of stromal and immune cells in malignant tumour tissues using expression data, *TIDE* tumor immune dysfunction and exclusion. *P < 0.05, **P < 0.01, ***P < 0.001.

We obtained data on mutation distribution and TMB based on the mutation data of the whole set. The top 20 genes with the highest mutation frequency in the two groups are shown in Fig. 8A,B. The TMB of patients was higher in the low-risk group than in the high-risk group (Fig. 8C). In addition, we performed correlation analysis to examine the association of signature with TMB and tumour stem cells, and the results suggested that the risk score was significantly negatively correlated with TMB, DNA stemness scores and RNA stemness scores (Fig. 8D–F). Furthermore, we identified whether TMB could predict the prognosis of patients with LSCC. According to the median value of TMB, patients were divided into the high- and low-TMB groups. Patients in the high-TMB group had a worse outcome than those in the low-TMB group (Fig. 8G). Moreover, a combination of the risk score and TMB effectively discriminated between patients with good and poor prognoses (Fig. 8H). To investigate differences in the immune status between the high- and low-risk groups, we computed immune, stromal and ESTIMATE scores and tumour purity using the ESTIMATE algorithm and evaluated immune cell infiltration and related functions via ssGSEA analysis (Fig. 9A). We found that the high-risk group had higher immune, stromal and ESTIMATE scores but lower tumour purity than that of the low-risk group (Fig. 9B–E). Importantly, both the number and functions of immune cells were elevated in the high-risk group than in the low-risk group (Fig. 9F,G). Given the significance of immune checkpoints to immunotherapeutic agents, we compared differences in the overall expression of 27 immune checkpoints. As shown in Fig. 9H, differences were observed in the expression levels of CD40LG, CTLA4, HAVCR2 and PDCD1 between the two groups. Furthermore, we used the TIDE database to investigate whether the signature could serve as an immunotherapeutic biomarker and found that patients in the low-risk group responded better to immunotherapy compared with patients in the high-risk group (Fig. 9I).

### Identification of potential compounds targeting the signature

Chemotherapy is a powerful therapeutic approach for patients with lung cancer, particularly among those with advanced TNM stages^30^. Therefore, we identified novel candidate drugs by calculating IC50 values of compounds based on the GDSC database. We identified 28 compounds with significant differences in IC50 values between the two groups (Fig. S1). These compounds exhibited greater sensitivity to the patients in the high-risk group than to patients in the low-risk group.

### Establishment of a prognostic nomogram

**Figure 10:**
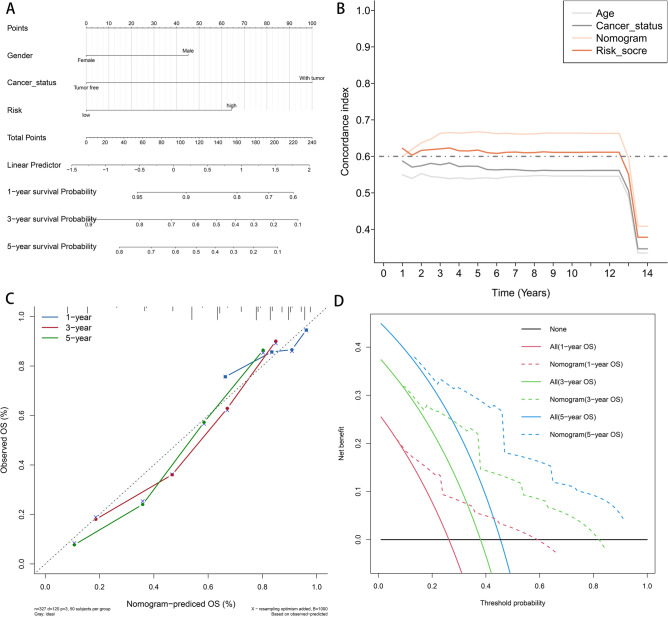
The nomogram model for the prediction of prognosis for the TCGA whole set. (**A**) Nomogram contained gender, cancer status and risk to predict survival. (**B**) Concordance index graph of nomogram in different years. (**C**) Calibration curve for nomogram in 1-, 3-, 5-year OS. (**D**) Decision curve analysis for nomogram in 1-, 3-, 5-year OS. *TCGA* The Cancer Genome Atlas, *OS* overall survival.

To determine the independent prognostic factors of LSCC, univariate and multivariate Cox regression analyses were conducted on the whole set. Univariate Cox regression analysis demonstrated that sex, T stage, cancer status and risk score were correlated with the overall survival rate of patients with LSCC (Table 2). However, multivariate Cox regression analysis revealed that only sex, cancer status and risk score had independent prognostic significance for LSCC (Table 2). Based on these independent prognostic factors, a nomogram was constructed to predict the 1-, 3- and 5-year OS of patients with LSCC (Fig. 10A). The C index of the nomogram for different years was always higher than that of other single independent prognostic factors, suggesting the nomogram could better predict the prognosis of LSCC (Fig. 10B). The calibration curve for predicting 1-, 3- and 5-year OS demonstrated a sufficient agreement between nomogram prediction and actual observation (Fig. 10C). In addition, the DCA curve revealed that the nomogram had higher net benefits for a range of threshold probabilities (Fig. 10D).

**Table 2:** Univariate and multivariate Cox analysis of the clinicopathological features and signature with OS.

Characteristics	Univariate Cox		Multivariate Cox	
	HR (95% CI)	*P* value	HR (95% CI)	*P* value
Age	1.217 (0.853–1.737)	0.278		
Gender	1.769 (1.249–2.505)	**0.001**	1.54 (1.082–2.194)	**0.017**
T	1.435 (1.042–1.976)	**0.027**	0.99 (0.708–1.385)	0.954
N	1.025 (0.785–1.339)	0.856		
M	2.066 (1.017–4.198)	0.045	1.783 (0.872–3.644)	0.113
Cancer status	2.915 (2.247–3.781)	** < 0.001**	2.664 (2.033–3.49)	** < 0.001**
Risk score	0.493 (0.376–0.646)	** < 0.001**	0.534 (0.404–0.707)	** < 0.001**

Significant value is given in bold.

### Validation of the expression of 11 pyroptosis-related lncRNAs

**Figure 11:**
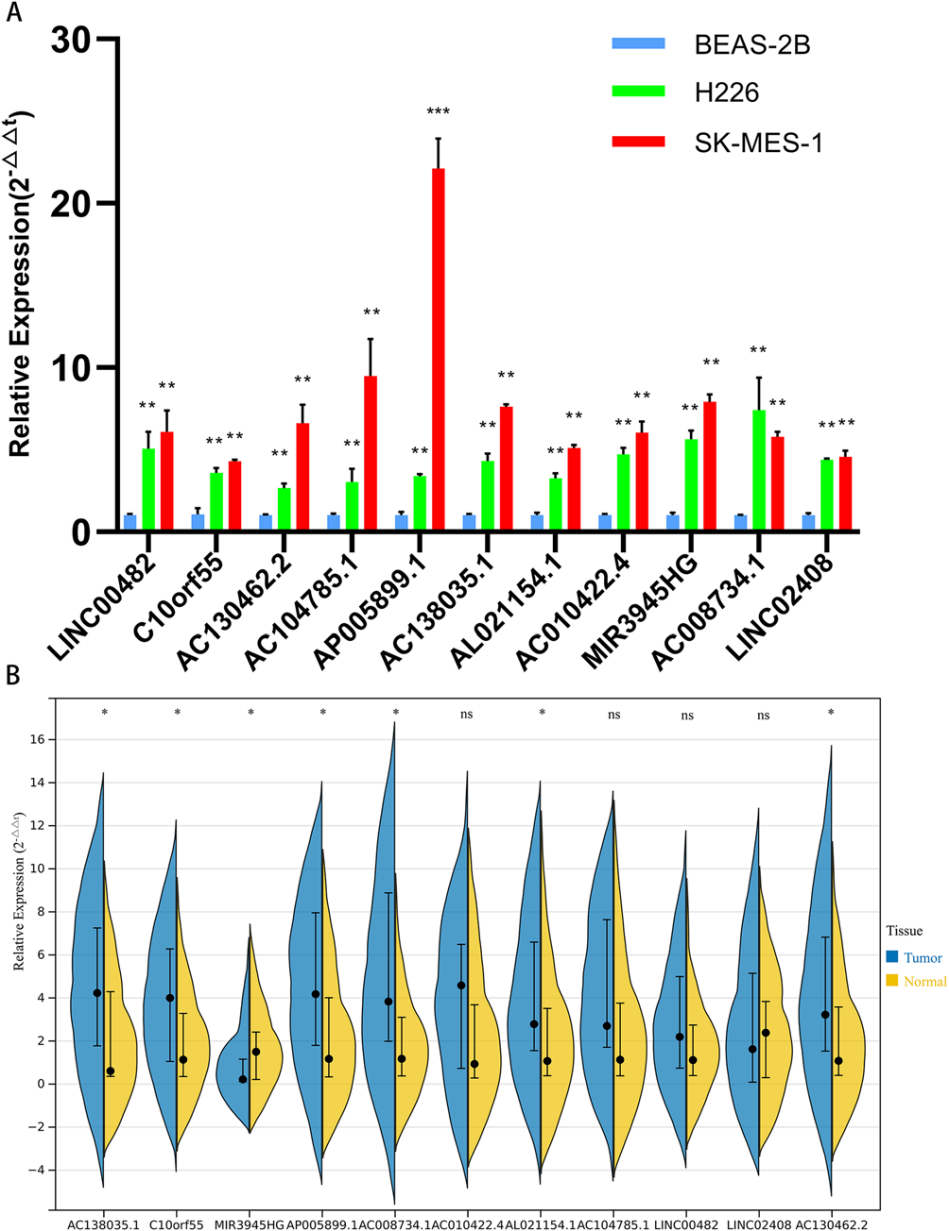
The expression validation of the lncRNAs in the signature. (**A**) Expression levels of 11 pyroptosis-related lncRNAs in the normal lung epithelial cell line BEAS-2B and two LSCC cell lines (A549, NCI-H1975) by RT-qPCR. (**B**) Expression levels of 11 pyroptosis-related lncRNAs in LSCC tissues and matched para-cancerous tissues by RT-qPCR. *lncRNA* long non-coding RNAs, *LSCC* lung squamous cell carcinoma, *RT-qPCR* quantitative real-time polymerase chain reaction. *P < 0.05, **P < 0.01, ***P < 0.001.

To evaluate the differences in the expression of the 11 lncRNAs that form the signature in various LSCC cell lines and clinical samples, we detect the expression levels of the 11 lncRNAs quantified by qRT-PCR. As shown in Fig. 11A, all lncRNAs were obvious highly expressed in the tumor cell lines. Besides, we found that AC138035.1, C10orf55, AP005899.1, AC008734.1, AL021154.1 and AC130462.2 were upregulated in tumour tissues than those in adjacent normal tissues, whereas MIR3945HG was downregulated (Fig. 11B). The results revealed that most of the expression of lncRNAs could be validated in LSCC cell lines and tissues.

## Discussion

LSCC is a common type of malignant tumour with a high burden of mortality, causing approximately 400,000 deaths worldwide every year^1^. In recent years, numerous studies have been conducted to explore the diagnosis, progression, prognosis and treatment of LSCC^31,32^. Some traditional tumor markers have been applied in screening, occurrence and prognosis prediction for LSCC^33^, including squamous cell carcinoma antigen (SCC), carcino-embryonic antigen (CEA), cytokeratins21-2 and neuron specific enolase (NSE). However, owing to the relatively low sensitivity and specificity of these biomarkers, the actual effectiveness in clinical settings is less satisfactory. The rapid development of high-throughput sequencing has provided valuable data for studying the mechanism of cancer, and studies are increasingly focussing on identifying biomarkers with non-coding RNAs to predict the prognosis and therapeutic efficacy of LSCC^34,35^.

Pyroptosis, a new paradigm of programmed cell death and a promising area of research, is functionally involved in diverse diseases such as atherosclerosis, neurodegeneration and tumours^20,36,37^. As a large and important group of non-coding RNAs, lncRNAs play a crucial role in different aspects of carcinogenesis and have emerged as a new type of biomarker in the diagnosis and prognosis of patients^38^. Several recent studies have investigated the regulatory effect of lncRNAs on pyroptosis. For example, the lncRNA MEG3 induces pyroptosis through activation of the NLRP3 inflammasome^39^. MEG3 knockdown suppresses the activation effect of DDP on NLRP3/caspase-1/GSDMD pathway-mediated pyroptosis and alters the inhibitory effect of DDP on tumour proliferation and metastasis in triple-negative breast cancer^40^. Xu et al. discovered that the lncRNA XIST can promote tumour growth and mediate drug-resistant behaviour in NSCLC by decreasing the nuclear transfer of SMAD2, thus inhibiting pyroptosis by suppressing the transcription of NLRP3^41^. Most studies on pyroptosis in cancer have focussed on coding RNAs but lack systematic analysis on the clinical value of pyroptosis-related lncRNAs for LSCC^42,43^. Therefore, it is necessary to construct a signature based on pyroptosis-related lncRNAs for predicting the prognosis and immunotherapy response and identifying potential target drugs in large cohorts.

In this study, we constructed and validated a novel and an efficient pyroptosis-related lncRNA prognostic signature for LSCC based on the TCGA dataset. Patients with LSCC were divided into the high- and low-risk groups based on the median risk score. The Kaplan–Meier survival curves revealed that the signature could distinguish between patients with a good prognosis and those with a poor prognosis in the training, test, whole and GSE81089 sets. The AUC values calculated from the ROC curves in the TCGA cohort suggested that the signature had a good predictive ability for OS of patients with LSCC. Furthermore, compared with the previously reported lncRNA-based signatures, the signature established in this study showed a better performance of survival prediction in the same LSCC cohort. These results supported the efficacy and robustness of the established signature. We further found that the signature was significantly associated with sex, N stage, cancer status and survival status. Moreover, the signature and relevant clinical features may be efficient and independent prognostic factors. In addition, a nomogram was established to predict the 1-, 3- and 5-year survival rates quantitatively and intuitively, which may offer net benefits in clinical settings. Besides, the expression of theses dysregulated lncNRAs in the signature was validated by qPCR in LSCC cell lines and tissues.

Among the lncRNAs used to establish the signature, LINC00482 is overexpressed in bladder cancer tissues and cells, and silencing LINC00482 may inhibit the proliferation, migration and invasion of bladder cancer by repressing the expression of MMP15 by targeting FOXA1^44^. Chen et al. found that patients with head and neck squamous cell carcinoma with high C10orf55 expression showed worse disease-free survival than that of patients with low C10orf55 expression^45^. It is worth mentioning that AC104785.1 was also identified as an autophagy-related lncRNA by a similar method, the expression of AC104785.1 was negatively correlated with bladder cancer prognosis^46^. This suggests that some lncRNAs may play a role in multiple programmed cell death but require further functional verification. To the best of our knowledge, other lncRNAs were identified for the first time. Therefore, more studies should be conducted to examine the clinical roles and underlying pyroptotic mechanisms of these novel lncRNAs in LSCC.

Successful treatment of LSCC is a significant challenge for modern medicine. With the rapid development of cancer treatment technology, both molecular therapy and immunotherapy hold great promise for the treatment of patients with NSCLC^47^. However, no significant breakthrough has been achieved in developing accurate indicators that can identify patients who may benefit from immunotherapy. TMB refers to the total number of somatic mutations in specific regions of a tumour genome^48^ and has emerged as a predictor of immunotherapy response in various types of cancer^49^. In this study, we reported that TMB values were higher in the low-risk group than in the high-risk group and were negatively correlated with risk scores. Whether used alone or in combination with risk scores, TMB can help to differentiate patients with LSCC with good prognosis from those with poor prognosis. Furthermore, the immune microenvironment can substantially interfere with the efficiency of immunotherapy^50^; therefore, we examined the immune status in different risk groups and found that the high-risk group had a higher number of infiltrating immune cells and stronger immune function than that of the low-risk group. In addition, significant differences were found in the expression of most immune checkpoints between the two groups. The TIDE algorithm is a computational framework designed to predict the immunotherapy response by stimulating the mechanisms of immune evasion used by tumours 26. In this study, the TIDE algorithm suggested that patients in the low-risk group had a better response to immunotherapy. Chemotherapy is an important treatment strategy for lung cancer, although targeted therapy and immunotherapy have currently become more popular^51^. We identified 28 novel candidate compounds targeting the signature from the GDSC project data. These findings indicated that the signature may help to identify effective immune markers and therapeutic targets for anti-tumour treatment.

However, this study has several limitations. First, some clinical information, such as operation methods, was not available in TCGA database, which may lead to bias and errors. Second, this research focused on retrospective analysis based on public databases and only was involved in expression validation. In addition, due to different properties for LSCC cell lines and small number for tissue samples, not all lncRNAs expressed levels were in agreement with results in the database. Therefore, prospective and independent datasets with large sample sizes are required to further confirm the pyroptosis-related lncRNA signature. Third, the underlying mechanism of lncRNAs in modulating pyroptosis in LSCC remains unclear. Despite these limitations, this study demonstrated the feasibility and effectiveness of the signature and suggested avenues for future studies on molecular mechanisms of lncRNAs and clinical studies on pyroptosis-related lncRNAs in LSCC.

## Conclusion

We identified pyroptosis-related lncRNAs and constructed a signature for predicting the prognosis of LSCC using large sample size. In addition, the signature identified not only novel chemotherapeutic drugs for adjuvant treatment but also patients who may be sensitive toward immunotherapy. Therefore, the pyroptosis-related lncRNA-based signature may serve as a potential clinical biomarker and therapeutic target for patients with LSCC.

## Supplementary Information

Supplementary Figure S1.

Supplementary Table S1.

Supplementary Table S2.

## Data Availability

The datasets generated and/or analyzed in the current study all obtained from public databases. This data can be found here: https://portal.gdc.cancer.gov/ and https://www.ncbi.nlm.nih.gov/geo/.

## References

[1] Siegel RL (2021). Cancer statistics, 2021. CA Cancer J. Clin..

[2] Herbst RS, Morgensztern D, Boshoff C (2018). The biology and management of non-small cell lung cancer. Nature.

[3] Lemjabbar-Alaoui H (2015). Lung cancer: Biology and treatment options. Biochim. Biophys. Acta..

[4] Lim SL (2018). Metabolic signatures of four major histological types of lung cancer cells. Metabolomics.

[5] Kovacs SB, Miao EA (2017). Gasdermins: Effectors of pyroptosis. Trends Cell Biol..

[6] Fink SL, Cookson BT (2006). Caspase-1-dependent pore formation during pyroptosis leads to osmotic lysis of infected host macrophages. Cell Microbiol..

[7] Man SM, Karki R, Kanneganti TD (2017). Molecular mechanisms and functions of pyroptosis, inflammatory caspases and inflammasomes in infectious diseases. Immunol. Rev..

[8] Wang L (2021). Structures and functions of the inflammasome engine. J. Allergy Clin. Immunol..

[9] Chu Q (2016). Pyroptosis is involved in the pathogenesis of human hepatocellular carcinoma. Oncotarget.

[10] Chen YF, Qi HY, Wu FL (2018). Euxanthone exhibits anti-proliferative and anti-invasive activities in hepatocellular carcinoma by inducing pyroptosis: Preliminary results. Eur. Rev. Med. Pharmacol. Sci..

[11] Clerc P (2018). Targeted magnetic intra-lysosomal hyperthermia produces lysosomal reactive oxygen species and causes Caspase-1 dependent cell death. J. Control Release.

[12] Johnson DC (2018). DPP8/DPP9 inhibitor-induced pyroptosis for treatment of acute myeloid leukemia. Nat. Med..

[13] Zhang T (2019). Transcription factor p53 suppresses tumor growth by prompting pyroptosis in non-small-cell lung cancer. Oxid. Med. Cell Longev..

[14] Teng JF (2020). Polyphyllin VI induces caspase-1-mediated pyroptosis via the induction of ROS/NF-κB/NLRP3/GSDMD signal axis in non-small cell lung cancer. Cancers (Basel).

[15] Mercer TR, Dinger ME, Mattick JS (2009). Long non-coding RNAs: Insights into functions. Nat. Rev. Genet..

[16] Hahne JC, Valeri N (2018). Non-coding RNAs and resistance to anticancer drugs in gastrointestinal tumors. Front. Oncol..

[17] Müller V (2019). Interplay of lncRNA H19/miR-675 and lncRNA NEAT1/miR-204 in breast cancer. Mol. Oncol..

[18] Derrien T (2012). The GENCODE v7 catalog of human long noncoding RNAs: Analysis of their gene structure, evolution, and expression. Genome Res..

[19] Karki R, Kanneganti TD (2019). Diverging inflammasome signals in tumorigenesis and potential targeting. Nat. Rev. Cancer.

[20] Xia X (2019). The role of pyroptosis in cancer: Pro-cancer or pro-"host"?. Cell Death Dis..

[21] Wang B, Yin Q (2017). AIM2 inflammasome activation and regulation: A structural perspective. J. Struct. Biol..

[22] Man SM, Kanneganti TD (2015). Regulation of inflammasome activation. Immunol. Rev..

[23] Hu J (2019). Systematic analysis identifies three-lncRNA signature as a potentially prognostic biomarker for lung squamous cell carcinoma using bioinformatics strategy. Transl. Lung Cancer Res..

[24] Zheng R (2021). Identification of a prognostic long noncoding RNA signature in lung squamous cell carcinoma: A population-based study with a mean follow-up of 3.5 years. Arch. Public Health.

[25] The Gene Ontology Consortium (2019). The Gene Ontology Resource: 20 years and still GOing strong. Nucleic Acids Res..

[26] Kanehisa M (2021). KEGG: Integrating viruses and cellular organisms. Nucleic Acids Res..

[27] Wu Z (2020). Identification of gene expression profiles and immune cell infiltration signatures between low and high tumor mutation burden groups in bladder cancer. Int. J. Med. Sci..

[28] Lebwohl D, Canetta R (1998). Clinical development of platinum complexes in cancer therapy: An historical perspective and an update. Eur. J. Cancer.

[29] Garnett MJ (2012). Systematic identification of genomic markers of drug sensitivity in cancer cells. Nature.

[30] Jiang P (2018). Signatures of T cell dysfunction and exclusion predict cancer immunotherapy response. Nat. Med..

[31] Jia S (2021). Transcriptome based estrogen related genes biomarkers for diagnosis and prognosis in non-small cell lung cancer. Front. Genet..

[32] Yang J (2021). miR-30a-5p suppresses lung squamous cell carcinoma via ATG5—Mediated autophagy. Aging (Albany).

[33] Foa P (1999). Tumour markers CEA, NSE, SCC, TPA and CYFRA 21.1 in resectable non-small cell lung cancer. Anticancer Res..

[34] Hou Z (2014). Long noncoding RNAs expression patterns associated with chemo response to cisplatin based chemotherapy in lung squamous cell carcinoma patients. PLoS ONE.

[35] Pan J, Huang Z, Xu Y (2021). m5C RNA methylation regulators predict prognosis and regulate the immune microenvironment in lung squamous cell carcinoma. Front. Oncol..

[36] Xu YJ (2018). Pyroptosis and its relationship to atherosclerosis. Clin. Chim. Acta.

[37] Pirzada RH, Javaid N, Choi S (2020). The roles of the NLRP3 inflammasome in neurodegenerative and metabolic diseases and in relevant advanced therapeutic interventions. Genes (Basel).

[38] Jin KT (2020). The role of long non-coding RNAs in mediating chemoresistance by modulating autophagy in cancer. RNA Biol..

[39] Zhang Y (2018). Melatonin prevents endothelial cell pyroptosis via regulation of long noncoding RNA MEG3/miR-223/NLRP3 axis. J. Pineal Res..

[40] Yan H (2021). Cisplatin induces pyroptosis via activation of MEG3/NLRP3/caspase-1/GSDMD pathway in triple-negative breast cancer. Int. J. Biol. Sci..

[41] Xu X (2020). Silencing of lncRNA XIST inhibits non-small cell lung cancer growth and promotes chemosensitivity to cisplatin. Aging (Albany).

[42] Ye Y, Dai Q, Qi H (2021). A novel defined pyroptosis-related gene signature for predicting the prognosis of ovarian cancer. Cell Death Discov..

[43] Shao W (2021). The pyroptosis-related signature predicts prognosis and indicates immune microenvironment infiltration in gastric cancer. Front. Cell Dev. Biol..

[44] Wang Y (2020). Silencing LINC00482 inhibits tumor-associated inflammation and angiogenesis through down-regulation of MMP-15 via FOXA1 in bladder cancer. Aging (Albany).

[45] Chen G (2021). PLAU promotes cell proliferation and epithelial-mesenchymal transition in head and neck squamous cell carcinoma. Front. Genet..

[46] Wan J (2021). Autophagy-related long non-coding RNA is a prognostic indicator for bladder cancer. Front. Oncol..

[47] Naylor EC, Desani JK, Chung PK (2016). Targeted therapy and immunotherapy for lung cancer. Surg. Oncol. Clin. N. Am..

[48] Allgäuer M (2018). Implementing tumor mutational burden (TMB) analysis in routine diagnostics—A primer for molecular pathologists and clinicians. Transl. Lung Cancer Res..

[49] Chan TA (2019). Development of tumor mutation burden as an immunotherapy biomarker: Utility for the oncology clinic. Ann. Oncol..

[50] Messenheimer DJ (2017). Timing of PD-1 blockade is critical to effective combination immunotherapy with anti-OX40. Clin. Cancer Res..

[51] Gottschling S (2012). Are we missing the target? Cancer stem cells and drug resistance in non-small cell lung cancer. Cancer Genomics Proteomics..

