# Supplementary figures and images for: Identification of pyroptosis-related long non-coding RNAs with prognosis and therapy in lung squamous cell carcinoma
Source: Sci Rep. 2022 Jul 1;12:11206. doi: 10.1038/s41598-022-15373-6 (PMC9249737; doi:10.1038/s41598-022-15373-6)

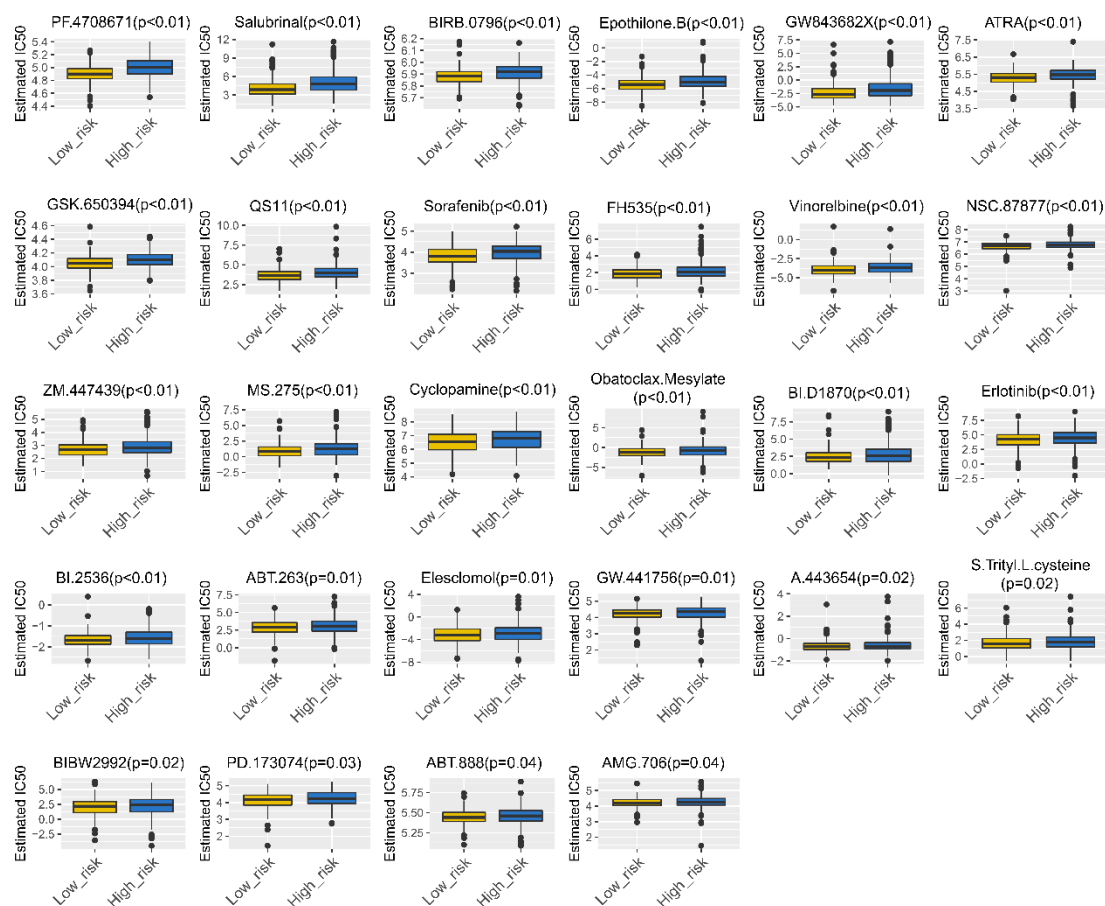

Figure S1: Boxplots of estimated IC<sub>50</sub> values of potential compounds in different groups.

Supplement: Supplementary file 1 — Supplementary Figure S1. [file 41598_2022_15373_MOESM1_ESM.pdf]
