# Supplementary material for: Identification of pyroptosis-related long non-coding RNAs with prognosis and therapy in lung squamous cell carcinoma
Source: Sci Rep. 2022 Jul 1;12:11206. doi: 10.1038/s41598-022-15373-6 (PMC9249737; doi:10.1038/s41598-022-15373-6)
Supplement: Supplementary file 2 — Supplementary Table S1. [file 41598_2022_15373_MOESM2_ESM.docx]

**Table S1.** The name of pyroptosis-related genes.

| AIM2 |
| --- |
| CASP1 |
| CASP3 |
| CASP4 |
| CASP5 |
| CASP6 |
| CASP8 |
| CASP9 |
| ELANE |
| GPX4 |
| GSDMA |
| GSDMB |
| GSDMC |
| GSDMD |
| GSDME |
| IL18 |
| IL1B |
| IL6 |
| NLRC4 |
| NLRP1 |
| NLRP2 |
| NLRP3 |
| NLRP6 |
| NLRP7 |
| NOD1 |
| NOD2 |
| PJVK |
| PLCG1 |
| PRKACA |
| PYCARD |
| SCAF11 |
| TIRAP |
| TNF |
